# Brace and Physiotherapeutic Scoliosis Specific Exercises (PSSE) for Adolescent Idiopathic Scoliosis (AIS) treatment: a prospective study following Scoliosis Research Society (SRS) criteria

**DOI:** 10.1186/s40945-022-00150-5

**Published:** 2022-11-01

**Authors:** Nikos Karavidas, Dionysios Tzatzaliaris

**Affiliations:** 1 Schroth Scoliosis & Spine Clinic, Athens, Greece; 2 Scoliosis Spine Laser Center, Athens, Greece

**Keywords:** Brace, PSSE, Scoliosis, Compliance, Scoliosis exercises

## Abstract

**Background:**

A growing scientific evidence for conservative treatment of AIS has recently proved that bracing is superior to natural history. Our aim was to investigate the effectiveness of a combined treatment with brace and PSSE for AIS.

**Methods:**

Prospective study, following SRS research inclusion criteria (> 10 years, 25^ο^ – 40^ο^, Risser 0–2, < 1-year post-menarche, no prior treatment). 102 consecutive patients (87 females-15 males, mean age 12.8 years, Risser 0.48, Cobb Thoracic 29.2^ο^, Lumbar 27.8^ο^) received treatment with Cheneau brace and PSSE. Average follow-up time was 26.4 months. A scale from A to C was used to evaluate compliance with brace and PSSE (A: full-compliant, B: partially compliant, C: non-compliant). 7 subjects dropped-out (6.8%), so finally 95 patients included for statistical analysis, using paired t-test.

**Results:**

Sixty-two patients (65.3%) remained stable, 22 improved > 5^ο^ (23.2%) and 11 progressed (11.5%). In-brace correction (IBC) was 49.7% for thoracic and 61.7% for lumbar curves. Analysis of progressed cases revealed that IBC (31.7% for thoracic and 34.4% for lumbar curves) and compliance (81.8% C for brace, 63.6% C for PSSE) was lower than average. Group A for treatment compliance (65.3%), showed significantly better results (70.9% stable, 29.1% improved, 0% progressed).

**Conclusion:**

A combination of bracing and PSSE can effectively treat AIS, according to SRS inclusion criteria. 88.5% of patients avoided progression > 5^ο^ and only 6.4% overpassed 40^ο^. IBC and compliance are the most important prognostic factors for successful treatment result. Early detection of AIS is also necessary for increased possibilities of effective conservative treatment.



## Introduction

According to Scoliosis Research Society (SRS) guidelines, brace treatment for Adolescent Idiopathic Scoliosis (AIS) is indicated for curves between 25^ο^ and 40^ο^ with remaining growth (Risser stage 0–3) [1]. Weinstein et al. with BrAIST (Bracing in Adolescent Idiopathic Scoliosis Trial) study in 2013 proved brace efficacy compared to natural history [2]. They used only brace without exercises and success rate was 72% for brace group compared to 48% in observational. A more recent meta-analysis by Zhang and Li (2019) included only Randomized Control Trials (RCT) comparing bracing with natural history and found that brace was superior to observation [3].

Physiotherapeutic Scoliosis Specific Exercises (PSSE) are curve pattern specific exercises, using 3D auto-correction, self-elongation, patient education and training for activities of daily life [4]. Recently, there is growing evidence about PSSE effectiveness for mild scoliosis < 25^ο^as a first step of treatment to halt progression and avoid bracing [4]. American Academy of Pediatrics (AAP), Pediatric Orthopedic Society of North America (POSNA), American Academy of Orthopedic Surgeons (AAOS) and SRS in their common Position Statement (2015) pinpointed that based on recent high-quality research, non-operative interventions (like bracing and PSSE) can decrease the likelihood of progression to the surgical threshold [5]. On the other hand, Fan et al. (2020) in a systematic review reported insufficient evidence for PSSE alone or combined with brace in reducing curve magnitude [6].

Some authors used a complete conservative treatment, by adding PSSE to standard of care or bracing and found significantly better results than bracing alone [7–10]. The aim of our study was to investigate the effectiveness of a combined non-operative treatment for AIS with brace and PSSE, in a population with high-risk of progression. Our hypothesis was that brace and PSSE can provide efficient conservative treatment for AIS.

## Methods

Our study design was prospective and performed under the approval of the Greek National Bioethics and Technoethics Commission, in agreement with the ethical standards as laid down in the 1964 Declaration of Helsinki. Level of Evidence was II according to Oxford Center for Evidence-Based Medicine (2011) [1]. Subject’s recruitment took place in our clinic, from April 2017 onwards. A research information sheet was given to the participants and a consent form was signed by their parents to allow the use of clinical data for research purpose. The data were collected from a prospective database. All subjects who fulfilled our entry requirements were screened from the initiation to the end of treatment and included for analysis, to avoid selection bias.

### Inclusion criteria

Our inclusion criteria strictly followed SRS research recommendations, so eligible for our study were all patients that had brace prescription for AIS, older than 10 years, with a Cobb angle between 25^ο^ and 40^ο^for at least one structural curve, at growth stage Risser 0–2, being less than 1-year post-menarche and having no previous treatment [1].

We included only patients with asymmetrical Cheneau braces, constructed by the same Orthotist who collaborated with our clinic, to standardize brace quality. Rigo classification for bracing was used by the main author who gave the proper instructions for brace fabrication to the Orthotist [11]. Patients with non-idiopathic scoliosis, or those wearing Boston brace/ Dynamic Derotational Brace (DDB) were excluded. Boston and DDB braces are symmetrical with posterior opening, so we tried to minimize potential confounders [12]. Cheneau braces have also been mentioned to be more compatible with Schroth method [8, 9, 13].

### X-ray measurement

X-rays were done at 3 stages. 1) before treatment, to define brace indication, 2) at 4 to 6 weeks with the brace fitted, to measure the in-brace correction and 3) at 1 year of treatment or when the brace was not fitted properly due to patient’s growth, always being out of brace for at least 24 h to eliminate brace correctional effect. The same process was followed for a second brace. All x-rays were measured independently by the Orthopedist who gave the brace prescription to the patient. To avoid measurement bias, we chose to use Orthopedic surgeon’s measurements for Cobb angle, because they were very experienced and totally blinded to our research. Cobb angle was digitally evaluated with Surgimap 2.3.2.1 software to increase validity. Only structural curves were measured, having both upper and lower end vertebrae tilted to the horizontal plane. Moreover, prior to “out of brace x-rays”, our patients were instructed to stay in a completely neutral position, to reduce misinterpretation of our results due to voluntary change to Schroth trained position [14].

### Treatment protocol

The brace prescription and the recommended wearing time was proposed by the Orthopedists. All patients were instructed to perform scoliosis specific exercises based on Schroth method [15], including exercises from Barcelona Scoliosis Physical Therapy School (BSPTS-Concept by Rigo), Schroth International Scoliosis Treatment (ISST) and Schroth Best Practice. BSPTS curve type classification was used for clinical evaluation and an individualized program of exercises was designed. Patients were instructed to perform them 5 times per week for approximately 30 min. One session every week took place in our clinic under supervision and the rest as a home exercise program.

### Compliance

We utilized a scale from A to C to estimate adherence to treatment protocol (A: full-compliant, B: partially compliant, C: non-compliant). Full compliance (A) for bracing was defined when brace wearing time was 90%-100% of recommended hours, partial compliance (B) for 70%-90% and poor compliance (C) for less than 70% of recommended hours. Similarly, for PSSE, Full compliance (A) was defined when the frequency was at least 5 days/week, partial compliance (B) for 3–4 days/week and poor compliance (C) for less than 3 days/week.

Compliance for brace and PSSE was self-reported by the patients and their parents. An independent compliance report form was filled from patients and parents and the mean value was used to increase reliability (Table 1). For brace wearing time they were asked to keep a daily diary and to provide us this form monthly. Similarly, for PSSE they kept a weekly diary and every month it was submitted to the authors. All the data were transferred to patient’s medical files and compared with the recommended brace wearing time and frequency of exercises, to estimate their compliance according to the scaling from A to C.

**Table 1:** The definition of compliance for brace and PSSE

**Brace Compliance A**	90%-100% of recommended hours	**PSSE Compliance A**	5 days/week or more
**Brace Compliance B**	70%-90% of recommended hours	**PSSE Compliance B**	3–4 days/week
**Brace Compliance C**	< 70% of recommended hours	**PSSE Compliance C**	< 3 days/week

### Outcome parameters

SRS and Society on Scoliosis Orthopedic and Rehabilitation Treatment (SOSORT) research guidelines recommend that non-operative studies should report primary patient-centered and secondary predictive outcome measures [1]. Therefore, our outcome parameters were a) curve progression, b) reaching surgical threshold (> 40^ο^), c) In-brace correction (IBC), d) compliance, e) SRS-22 questionnaire with 4 categories (function, pain, self-image, mental health) and f) Angle Trunk Rotation (ATR) by scoliometer. Curve progression or improvement was characterized as a Cobb angle’s change > 6^ο^ for at least one structural curve [1]. SRS-22 questionnaire was given to the participants at the start and end of brace treatment [16]. Trunk rotation was regularly measured by our clinic’s physiotherapists (blinded to this research) to detect possible changes that might affect treatment plan (brace pressures, exercise strategy etc.), but the start and end values were finally analyzed.

Our study setting did not allow us to use an actual control group, either without treatment due to ethical reasons or with standard of treatment (brace alone without PSSE), as every patient in our clinic is generally instructed to follow scoliosis specific exercises when there is brace indication. However, for a subsequent analysis about statistical and clinical significance a control group was used by taking a sub-group of our subjects, excluding only those with A compliance in PSSE, in the assumption that partial and poor compliance with exercises is more representative to treatment with brace alone. Another statistical analysis was made to illustrate the effect of compliance and IBC on treatment result.

### Statistical analysis

Power sample size calculation (95% confidence interval—CI), based on previously published related literature, performed to determine the minimum sample for a sufficient statistical power (80%) for a moderate (0.50) to large (0.80) size effect, 85 participants were needed. The main outcome for the previous studies was to avoid progression and Negrini et al. (2009) [7] used a sample of 48 participants, Schreiber et al. (2015) [8] included 50 patients and Kwan et al. (2017) [10] 48 patients, while other authors [6] that used only exercises had at maximum 110 participants. We performed both Intention to Treat Analysis by including all dropouts as failures, and Efficacy Analysis by including only results without drop-outs. Paired t-test was used for statistical analysis (*p*≤ 0.05) with SPSS 26.0 software. Strengthening the Reporting of Observational Studies in Epidemiology (STROBE) statement was implemented to enhance methodological quality [17].

## Results

In total, 102 consecutive patients followed treatment with Cheneau type brace and PSSE. Mean age was 12.8 years (10.2 to 14.6), Risser sign 0.48 (0 to 2), Cobb thoracic 29.2^ο^ (25^ο^ to 40^ο^), Cobb Lumbar/Thoracolumbar 27.8^ο^ (25^ο^ to 40^ο^), ATR Thoracic 9.3^ο^ (4^ο^ to 15^ο^) and ATR Lumbar 7.4 (3^ο^ to 13^ο^). Mean follow-up time was 26.4 (12.3 to 41.2) months.

Based on the Lonstein formula [18] (Cobb angle – 3 X Risser sign / chronological age), the mean progression factor for our group before starting treatment was 2.17 for thoracic and 2.06 for lumbar curves, which corresponds to approximately 80% risk of progression.

### Drop-outs

Seven subjects (6.8%) withdrew from the study, so they were not included in the main analysis, but in a secondary considering all dropouts. We tried to address this loss to follow-up by contacting the participants to report the reasons (2 poor treatment adherence, 2 financial issues, 2 relocations and 1 surgical treatment). So, 95 patients were initially analyzed and completed follow-up, as Efficacy Analysis.

### Curve progression

**Fig. 1:**
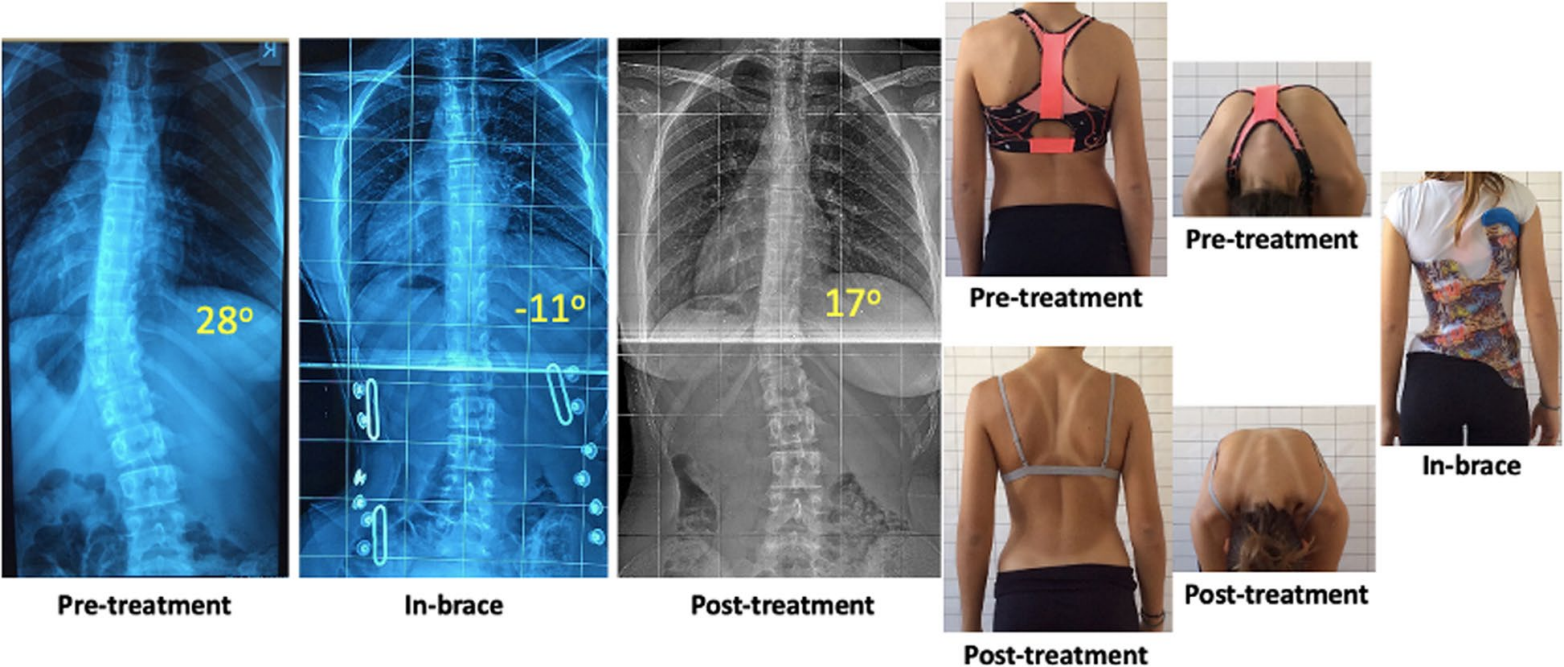
AIS Patient treated with brace and PSSE. Cobb angle was improved from 28^ο^ to 17^ο^. Trunk rotation (ATR) and pelvic symmetry were also significantly improved after treatment

Regarding curve progression our results showed that 62 patients (65.3%) remained stable, 22 improved > 5^ο^ (23.2%) and 11 progressed > 5^ο^ (11.5%). Post-intervention average Cobb Thoracic was 28.3^ο^ (10^ο^ to 51^ο^, 95% CI -0.45 to 2.06) and Cobb Lumbar/Thoracolumbar 26.1^ο^ (14^ο^ to 39^ο^, 95% CI 0.74 to 2.71) (Fig. 1). The mean difference was statistically significant for Lumbar Cobb (*p* = 0.0008) but not for Thoracic Cobb (*p* = 0.21). Only 6 patients (6.4%) overpassed the surgical indication range of 40^ο^, and 1 patient (1.1%) exceeded 50^ο^. In-brace correction (IBC) was 49.7% for thoracic and 61.7% for lumbar curves.

### Compliance

Our group in total revealed good compliance rate, having 65.3% with A-A (brace and PSSE respectively), 12.6% A-B, 3.1% A-C, 4.3% B-A, 3.1% B-B, 2.1% B-C, 0% C-A, 2.1% C-B and 7.4% C–C (Table 2).

**Table 2:** Compliance with brace and PSSE

	**Compliance** **PSSE A**	**Compliance** **PSSE B**	**Compliance** **PSSE C**	**Total**
**Compliance Brace A**	62 (65.3%)	12 (12.6%)	3 (3.1%)	77 (81%)
**Compliance Brace B**	4 (4.3%)	3 (3.1%)	2 (2.1%)	9 (9.5%)
**Compliance Brace C**	0 (0%)	2 (2.1%)	7 (7.4%)	9 (9.5%)
**Total**	66 (69.6%)	17 (17.8%)	12 (12.7%)	95 (100%)

### Quality of life—Aesthetics

SRS-22 overall questionnaire score before treatment was 81.4 (53 to 95) with sub-domains pain 22.3 (16 to 25), mental health 18.3 (9 to 25), self-image 19 (7 to 24) and function 22 (12 to 25). Post-treatment SRS-22 questionnaire overall score was 83.6 (58 to 98, 95% CI -2.65 to -1.39), pain 22.5 (17 to 25, 95% CI -0.5 to 0.09), mental health 18.9 (11 to 25, 95% CI -0.77 to -0.16), self-image 20.4 (11 to 24, 95% CI -2.18 to -1.00) and function 22.5 (14 to 25, 95% CI -0.96 to -0.28). (Table 3) The average difference was statistically significant for overall score (*p* = 0.0001), mental health (*p* = 0.003), self-image (*p* = 0.0001) and function (*p* = 0.0006), but not for pain (*p* = 0.17). Mean ATR measured by scoliometer was also significantly improved for both Thoracic (from 9.3 to 7.1^ο^, *p* = 0.002) and Lumbar curves (from 7.4^ο^ to 5.1^ο^, *p* = 0.001).

**Table 3:** SRS-22 questionnaire scores, before and after treatment (statistical significance)

**Pain pre-treatment**	22.3	**Pain** **post-treatment**	22.5	***p* = 0.17**
**Mental health pre-treatment**	18.3	**Mental health post-treatment**	18.9	*p* = 0.003*
**Self-image pre-treatment**	19	**Self-image** **post-treatment**	20.4	*P* = 0.0001*
**Function pre-treatment**	22	**Function** **post-treatment**	22.5	*P* = 0.0006*
**Total SRS-22 pre-treatment**	66 (69.6%)	**Total SRS-22** **post-treatment**	83.6	*P* = 0.0001*

### Sub-sequent analysis

Another analysis was done to compare the treatment result of the whole group with the result of partially and non-compliants with PSSE (B and C), using this sub-group of 29 patients as control. IBC was not statistically different compared to total group (47.7% for Thoracic and 59.3% for Lumbar, *p* = 0.12). 20 subjects remained stable (68.9%), 4 improved (13.8%) and 5 progressed (17.3%). The rate of progression (17.3%) was significantly more compared to the whole group (11.5%) (*p* = 0.03).

**Fig. 2:**
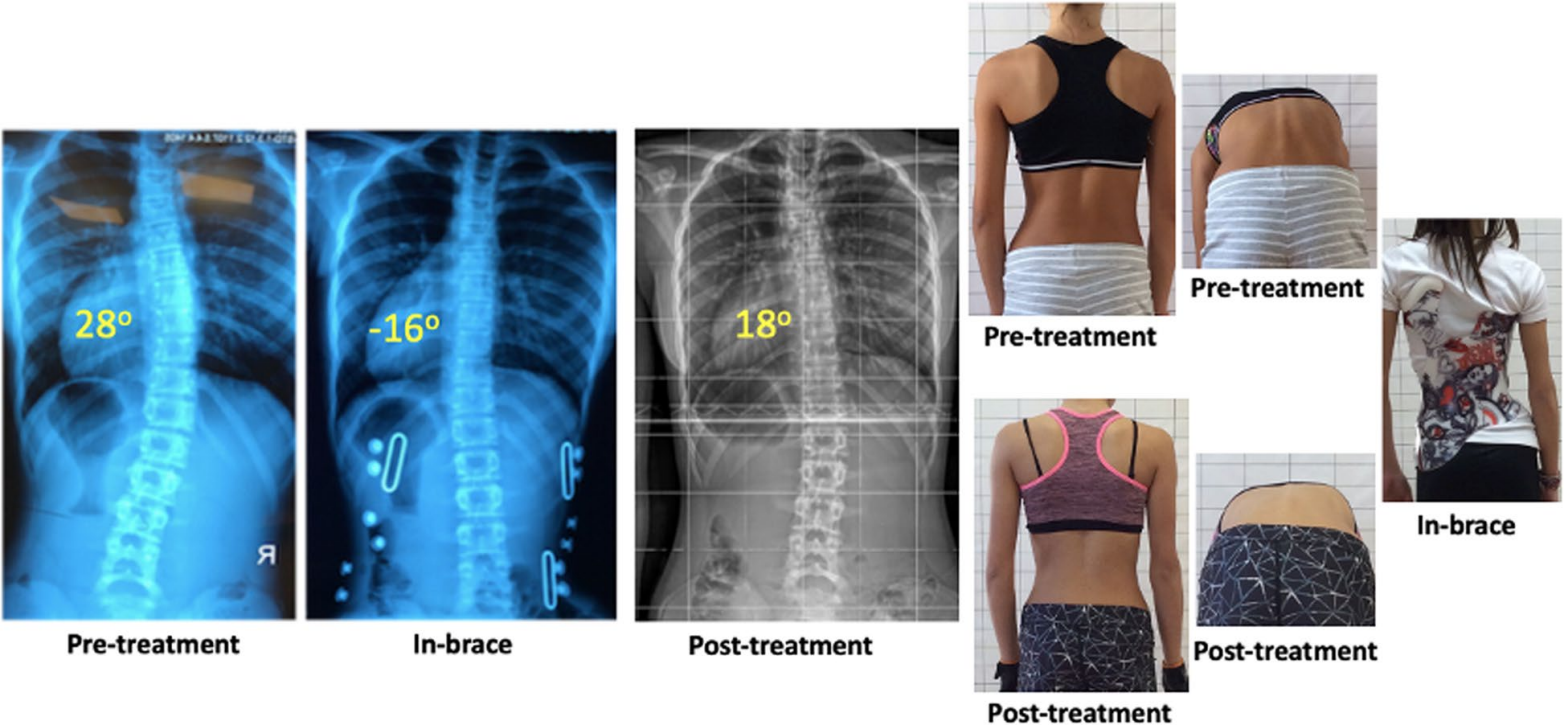
Radiological and clinical improvement in an adolescent with AIS, after brace and PSSE therapy

A further analysis was made for the A-A compliant group (brace and PSSE respectively). 62 subjects (65.3%) showed full compliance with brace and PSSE. 44 (70.9%) remained stable, 18 (29.1%) improved and 0 (0%) progressed (Fig. 2). The rate of progression (0%) was significantly less compared to the whole group (*p* = 0.002). We also analyzed our results by including the subjects that lost in follow-up (7 subjects, 6.8%) as the worst-case scenario, considering all of them as progressive cases. In this Intention to Treat analysis, our progression rate was increased to 17.6% (18 out of 102 subjects).

## Discussion

A complete non-operative treatment with a combination of bracing and PSSE can effectively treat AIS in adolescents with high risk of progression. 88.5% of participants avoided progression (65.3% stable, 23.2% improved), strictly following SRS research inclusion criteria. 6 patients (6.4%) exceeded 40^ο^ and 1 (1.1%) 50^ο^ at the end of treatment. In a worst-case scenario analysis, considering all dropouts as failed (ITTA), our success rate was still 82.4%. According to the Lonstein formula [18], the mean progression factor at the endpoint of our study was minimized to 1.24 for thoracic and 1.09 for lumbar curves, which corresponds to a risk of progression less than 20%.

### Comparability with other studies

Our results are comparable to previous studies with brace and PSSE, following SRS inclusion criteria. Negrini et al. (2009) [7] included 48 patients and presented 96% of successful treatment, with 0% reaching 45^ο^ or more. They combined Lyon or Sforzesco-SPoRT brace with Scientific Exercises Approach to Scoliosis (SEAS). Kwan et al. (2017) [10] combined Schroth with Boston brace, having 24 subjects and reported 79% success rate. The superiority of our results can be explained by the different brace design and better compliance, as we reported 81% A brace compliance compared to 70.8% good compliance in their study.

Other studies used SRS criteria for bracing without PSSE, having a success rate ranging from 67.7% to 98.5% [2, 19–23]. Ovadia et al. (2012) [19] used Rigo System Cheneau (RSC) brace, having 16.2% progression in a retrospective study with 93 patients, while Kuroki et al. (2015) [20] used Osaka Medical College (OMC) brace, having 32.3% progression. Aulisa et al. (2015) [21] reported 98.5% of successful treatment, but they included only single thoracic curves and provided no analysis for dropouts (16.7%). Pasquini et al. (2016) [22] reported 8% progression in all cases and 17% including dropouts as failed in a retrospective study with 37 participants. Weiss et al. (2019) [23] used Gensingen brace in a small sample of 28 patients, having 14.3% progression.

BrAIST study [2] defined successful treatment as end-result less than 50^ο^ and reported 72% of success rate, with an average brace wearing time of 11.8 h/day, while Weiss et al. (2019) [23] had 92.9% success rate with 20.3 average hours/day. In our study only 1 patient (1.1%) overpassed 50^ο^. Compliance and in-brace correction are the most important predictive factors for brace success [24]. Our study revealed good overall compliance, 81% was A for bracing and 70.5% for PSSE and this can be potentially attributed to the multi-professional approach in our clinic. A-A compliance group had no progression (0%), which confirms the necessity of adherence to brace wearing time and PSSE. In-brace correction was much more than 30% (49.7% for thoracic and 61.7% for lumbar curves), which is considered as a minimum for good prognosis [24].

**Fig. 3:**
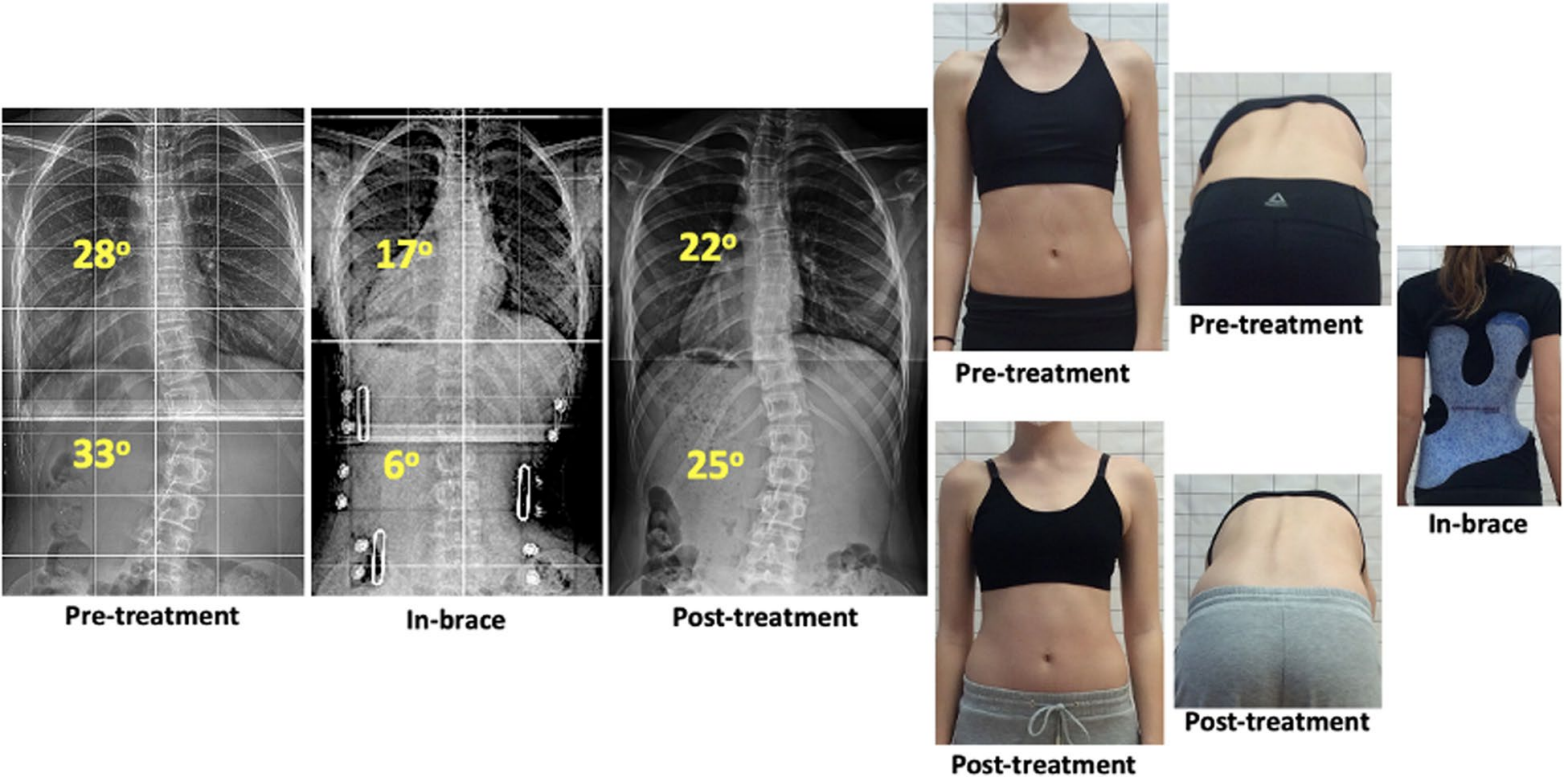
AIS Patient treated with brace and PSSE. Cobb angle was improved from 28^ο^ to 22^ο^ for thoracic curve and from 33^ο^ to 25^ο^ for thoracolumbar curve. Trunk rotation (ATR) and pelvic symmetry were also significantly improved after treatment

Our combined non-operative treatment also improved aesthetics and quality of life aspects, which are described as primary patient-centered outcomes by SRS and SOSORT research guidelines [1]. ATR was significantly improved for thoracic and lumbar curves (Fig. 3). SRS-22 scores and all sub-domains, except pain, achieved statistically significant improvement. Pain is generally not common in AIS, the main issues are mental health and self-image, which received the lower scoring in our sample and were markedly better post-treatment. The clinical relevance of our research was to maintain average Cobb angle post-treatment below 30^ο^, which seems to crucially decrease the risk of progression in adult life [18].

Taking the less compliant subjects with exercises (B and C) as a control group to compare with the whole group, we intended to identify the importance of PSSE to be added in the standard of conservative AIS treatment. Progression rate was remarkably higher (17.3%) in control group than overall (11.5%). Our outcomes regarding the importance of PSSE addition to treatment protocol agree with previous publications [7–10, 24]. Early detection of scoliosis is critical for prognostic value, as the success rate for braced patients > 40^ο^ in a recent study by Verhofste et al. (2020) was only 42% [25].

### Strengths

Our study had some important strengths in contrast to previously published research. We used a large sample size of 95 patients, after a power calculation, when other authors included notably less [7, 10, 20, 22]. Treatment was at high risk of progression, as we followed SRS inclusion criteria. We also made efforts to reduce selection and measurement bias by using a prospective database and blinding of assessors. Our outcomes can be generalized to adolescent population suffering from AIS, during the rapid growth.

### Limitations

However, several limitations could be recognized as well. This was an ongoing study and the follow-up stopped at the end of brace therapy. No data can be provided yet for the long-term maintenance of treatment effect. Another disadvantage was the lack of brace sensors to reliably evaluate compliance, so there might be an overestimation. We did not have a separate control group of bracing alone, therefore not fully compliant patients with PSSE were served as a control group in our secondary analysis.

## Conclusions

In conclusion, a complete conservative treatment with brace and PSSE achieved a success rate of 88.5% in a sample of 95 patients with Adolescent Idiopathic Scoliosis at peak of growth with relatively high risk of progression. Only 6.4% passed over 40^ο^ and 1.1% over 50% at the end of treatment. Non operative treatment with brace and scoliosis specific exercises, having adequate compliance and in-brace correction, can significantly reduce the likelihood of progression and reaching surgical indication range. Our treatment protocol also improved aesthetics, trunk rotation, mental health, and function in adolescents with scoliosis.

## Data Availability

The authors confirm that the data supporting the findings of this study are available within the article.

## Abbreviations

AAOS: American Academy of Orthopedic Surgeons
AAP: American Academy of Pediatrics
AIS: Adolescent Idiopathic Scoliosis
ATR: Angle Trunk Rotation
BrAIST: Bracing in Adolescent Idiopathic Scoliosis Trial
BSPTS: Barcelona Scoliosis Physical Therapy School
CI: Confidence Interval
DDB: Dynamic Derotational Brace
IBC: In-Brace Correction
ISST: International Schroth Scoliosis Treatment
OSM: Osaka Medical College
POSNA: Pediatric Orthopedic Society of North America
PSSE: Physiotherapeutic Scoliosis Specific Exercises
RCT: Randomized Control Trial
RSC: Rigo System Cheneau
SOSORT: Society on Scoliosis Orthopedic and Rehabilitation Treatment
SRS: Scoliosis Research Society
STROBE: Strengthening the Reporting of Observational Studies in Epidemiolog
